# Mass Media Exposure and Women’s Household Decision-Making Capacity in 30 Sub-Saharan African Countries: Analysis of Demographic and Health Surveys

**DOI:** 10.3389/fpsyg.2020.581614

**Published:** 2020-10-28

**Authors:** Abdul-Aziz Seidu, Bright Opoku Ahinkorah, John Elvis Hagan, Edward Kwabena Ameyaw, Eric Abodey, Amanda Odoi, Ebenezer Agbaglo, Francis Sambah, Vivian Tackie, Thomas Schack

**Affiliations:** ^1^Department of Population and Health, University of Cape Coast, Cape Coast, Ghana; ^2^College of Public Health, Medical and Veterinary Sciences, James Cook University, Townsville, QLD, Australia; ^3^The Australian Centre for Public and Population Health Research, Faculty of Health, University of Technology Sydney, Ultimo, NSW, Australia; ^4^Department of Health, Physical Education and Recreation, University of Cape Coast, Cape Coast, Ghana; ^5^Neurocognition and Action-Biomechanics Research Group, Faculty of Psychology and Sport Sciences, Bielefeld University, Bielefeld, Germany; ^6^Department of Education and Psychology Studies, University of Cape Coast, Cape Coast, Ghana; ^7^Centre for Gender Research Advocacy and Documentation, University of Cape Coast, Cape Coast, Ghana; ^8^Department of English, University of Cape Coast, Cape Coast, Ghana

**Keywords:** household decision-making capacity, mass media, sub-Saharan Africa, women’s health, public health, demographic and health survey

## Abstract

**Background:**

Women’s household decision-making capacity is an essential component of their empowerment which include decisions related to personal health care, large household purchase and family visitations. Despite research evidence acknowledging mass media’s influences on women’s empowerment, including their ability to take household decisions, empirical data through multi-country comparison on mass media exposure and women’s decision making capacity are sparse. This study sought to assess the association between exposure to mass media (television, radio and newspaper/magazine) and women’s household decision-making capacity in 30 countries in sub-Saharan Africa (SSA).

**Materials and Methods:**

Data from current Demographic and Health Surveys (DHS) conducted in 30 countries in SSA from January 1, 2010 to December 31, 2016 were used. Binary Logistic Regression analysis was used to assess the association between mass media exposure and women’s household decision-making capacity in SSA. Results were presented using crude odds ratios (COR) and adjusted odds ratios (AOR).

**Results:**

Women who watched television almost every day had higher capacity to take household decisions, compared to those who did not watch television at all. Women who read newspaper/magazine less than once a week were less likely to take household decisions compared to those who never read newspaper/magazine. However, there was no association between exposure to radio and household decision-making capacity. Regarding the covariates, age, level of education, wealth index, occupation, and parity showed significant associations with women’s household decision-making capacity.

**Conclusion:**

Findings stressed the positive contribution of mass media in enhancing women’s household decision-making capacity in SSA. Viewing television, a model of mass media, is a very powerful conduit to enhance the household decision-making capacity of women. The use of mass media, especially television in communicating the relevance and ways of achieving household decision-making capacity for all women in SSA is paramount and perhaps, in other low and middle-income countries of the world. Interest groups that require greater attention are women with less exposure to television as well as women in their early reproductive age, the poor, women who are not working and rural residents.

## Introduction

Research has shown that improved women’s status comes with some benefits. For example, it leads to reduction in fertility. Hence, policymakers, especially those in low and middle-income countries, are beginning to pay particular attention to issues relating to women empowerment, which includes their household decision-making capacity (Upadhyay et al., 2014; Prata et al., 2017; Dasgupta, 2019). Generally, women’s empowerment concerns women’s aptitude to have access to various aspects of development, including “health, education, earning opportunities, rights, and political participation” (Duflo, 2012), and their capability in making free choices (Torp, 2012). Essentially, socio-economic concerns, including education and freedom of movement, have often been considered as benchmark for the measurement of women empowerment (Afridi, 2010).

Women’s household decision-making capacity is an essential component of their empowerment and include “decision on personal health care,” “decision on large household purchase,” and “decision on visits to family or relatives” (Acharya et al., 2010). As a concept, decision-making is a process that begins with problem identification, data collection and gathering, analysis of data, findings, selecting an appropriate and most suitable solution from the alternative solutions, and evaluation of the process (Reinicke et al., 2000). The ability of women to take household decisions that affect their health, family and social life is essential for their survival in society. For instance, a woman’s capacity to take health-care decisions is extremely important for better maternal and child health outcomes (Ahinkorah et al., 2018).

Despite studies indicating the negative impact of media on the body image satisfaction of women (Scirrotto Drames, 2016; Uchôa et al., 2019), all over the world, media has been considered as a powerful vehicle for bringing women’s rights issues to the attention of a wider public, galvanizing action on the streets of cities around the world and encouraging policy makers to step up commitments to gender equality (Loiseau and Nowacka, 2015). Narayana and Ahamad (2016) revealed that media has a great potential for the empowerment of women by increasing their participation and access of women to expression and decision-making. Social media has proven potential for mobilizing attention and accountability to women’s rights, and challenging discrimination and other stereotypical behaviors against them (Loiseau and Nowacka, 2015).

Previous researches have revealed that mass media influences women empowerment, including their ability to take household decisions (Jensen and Oster, 2009; Ting et al., 2014; Dasgupta, 2019). However, such studies have largely, often focused on telenovelas and radio programs and their influence on women’s child-naming practices (La Ferrara et al., 2012), use of prenatal services (Ghosh, 2006), and participation in social groups (Olken, 2009). Dasgupta (2019), for instance, notes that mass media exposure is likely to change some socio-cultural norms such as gender stereotyping especially in low-literate areas. In such areas, through mass media, gender stereotyping can be challenged by revealing information pertaining to some empowered women in such settings (Jesmin and Amin, 2017). Such information, which may include empowerment opportunities (e.g., educational and entrepreneurial training) available to women (Bhushan and Singh, 2014), is likely to positively affect women’s attitudes towards socio-cultural norms, which may cumulatively lead to their household decision-making capacity. However, none of the previous studies examined the association between mass media exposure and the household decision-making capacity of women in sub-Saharan Africa (SSA). Additionally, previous studies also did not look at women’s household decision-making capacity comprehensively from their decision on personal healthcare, large household purchase and visits to family or relatives. Apart from mass media exposure, studies have also found associations between some socio-demographic determinants (e.g., age, education) and household decision making capacity (Head et al., 2015; Akram, 2018; Yount et al., 2018). For example, women with higher education are more likely to take household decisions (Krause et al., 2016; Narayana and Ahamad, 2016; Sell and Minot, 2018). Wealth (Akram, 2018), occupation (Head et al., 2015; Salem et al., 2017) and place of residence have also been found to be associated with the capacity of a woman to take household decisions (Sharma and Shekhar, 2015; Sell and Minot, 2018). Relatedly, these factors may interplay with mass media exposure to influence a woman’s household decision making capacity.

The present study seeks to contribute to literature in this area of study by assessing the association between mass media exposure (radio, television and newspaper/magazine) and women household decision-making capacity in 30 countries in SSA, paying particular attention to “decision on personal healthcare,” “decision on large household purchase,” and “decision on visits to family or relatives.” Based on the objective of the study, it was hypothesized that women who are exposed to mass media (radio, television, and newspaper) would be more likely to have the capacity to take household decisions compared to those who are not exposed to mass media. This is desirable because previous studies (Jensen and Oster, 2009; Ting et al., 2014; Dasgupta, 2019) were done in India, a geographical setting where the socio-cultural environments might be different from the SSA context. Notably, women’s household decision making in India may differ from that of SSA. Again, while majority of Indians may access information from the internet, that may not be the case in most SSA countries where access to internet has been found to be low (Guerriero, 2015; Igwe, 2019). A Previous study in SSA found that only 12% of women have the capacity to make household decisions (Ahinkorah et al., 2018).

## Materials and Methods

### Source of Data

The study utilized pooled data from current Demographic and Health Surveys (DHS) conducted between January 1, 2010 and December 31, 2016 in 30 countries in SSA (see Table 1). DHS is a multi-country survey collected every 5-year period across low- and middle-income countries. The survey is representative of each of these countries. The DHS survey employs stratified two-stage sampling technique in order to ensure national representativeness (Aliaga and Ren, 2006). As described in detail previously (Mejía-Guevara et al., 2019), the first-stage constitutes the development of a sampling frame consisting of a list of primary sampling units (PSUs) or enumeration areas (EAs) which cover participation in entire country and are usually developed from the available latest national census. Each PSU or EA is further subdivided into standard size segments of about 100–500 households per segment. Then, a sample of predetermined segments is selected randomly with probability proportional to the EA’s measure of size (number of households in EA). The second stage involves the DHS survey personnel selecting households systematically from a list of previously enumerated households in each selected EA segment. In-person interviews are conducted in selected households with the target populations: women aged 15–49 and men aged 15–64. The number of selected households per EA is variable which ranges from 30 to 40 households/women per rural cluster and from 20 to 25 households per urban cluster (Mejía-Guevara et al., 2019). Three questionnaires were used for the data collection: a Household Questionnaire, a Women’s Questionnaire, and a Men’s questionnaire. The surveys were done at different times due to the variations in the starting points of the DHS in the various countries. The sample frame usually excludes nomadic and institutional groups such as prisoners and hotel occupants. As evident in other studies combining the DHS in SSA (Chol et al., 2019; Darteh et al., 2019; Mejía-Guevara et al., 2019), the starting points of the data surveys are different, although this procedure does not defeat the ability to compare the DHS among the countries due to the commonality in protocol and instruments used. Permission to use the data set was sought from MEASURE DHS. The data set is available to the public at https://dhsprogram.com/data/available-datasets.cfm. The sample size for the study was 47,135 women in unions who had complete cases on the variables of interest (see Table 1). These women were considered because the decision making capacity variables were only applicable to women in unions.

**TABLE 1:** Sample.

**Country**	**Weighted Frequency**	**Weighted Percentage**
Angola	5,262	11.2
Burkina Faso	522	1.1
Benin	1,928	4.1
Burundi	2,327	4.9
Congo DR	3,036	6.4
Congo	4,219	9.0
Côte d’Ivoire	2,153	4.6
Cameroon	904	1.9
Ethiopia	135	0.3
Gabon	2,468	5.2
Ghana	1,257	2.7
Gambia	216	0.5
Guinea	330	0.7
Kenya	1,046	2.2
Comoros	24	0.1
Liberia	2,814	6.0
Lesotho	143	0.3
Mali	196	0.4
Malawi	1,226	2.6
Mozambique	1,615	3.4
Nigeria	939	2.0
Namibia	2,782	5.9
Rwanda	2,380	5.1
Sierra Leone	1,239	2.6
Senegal	309	0.7
Chad	332	0.7
Togo	1,143	2.4
Uganda	4,814	10.2
Zambia	958	2.0
Zimbabwe	418	0.9
Total	47,135	100.0

### Definition of Variables

#### Dependent Variable

The dependent variable for the study was “women’s household decision-making capacity” which was derived from three questions “decision on personal health care,” “decision on large household purchase,” and “decision on visits to family or relatives.” These response categories were recoded as “not alone = 0” and “alone = 1”). An index was created with all the “yes” and “no” answers with scores ranging from 0 to 3. The score 0 and 1 were labeled as “less capacity” whereas 2 and 3 were labeled as “more capacity.” A dummy variable was generated with ‘0’ score being married women who were less empowered and ‘1’ if married women were more empowered. The inclusion criteria were all women in unions (15–49) who had answered these questions.

#### Explanatory Variables

The key explanatory variable was mass media exposure (radio, newspaper/magazine, and television). These variables were chosen from three questions derived from the DHS. These questions are: “Do you watch television almost every day, at least once a week, less than once a week or not at all? Do you read a newspaper or magazine at least once a week, less than once a week or not at all? Do you listen to the radio at least once a week, less than once a week or not at all?” The responses were: Not at all, less than once a week and at least once a week. Details of these variables and their coding are described in Table 2.

**TABLE 2:** Variables description and coding.

**No**	**Variable**	**Description/Question**	**Coding**
**Outcome variable**			
1.	Women’s household decision making capacity	The dependent variable for the study was “women’s household decision-making capacity” which was derived from three questions “decision on personal health care,” “decision on large household purchase” and “decision on visits to family or relatives	0 = Less capacity 1 = More capacity
**Explanatory/independent variables**			
2.	Frequency of reading newspaper or magazine	Do you read a newspaper or magazine at least once a week, less than once a week or not at all?	1 = Not at all 2 = Less than once a week 3 = At least once a week
3.	Frequency of watching television	Do you watch television at least once a week, less than once a week or not at all?	1 = Not at all 2 = Less than once a week 3 = At least once a week
4.	Frequency of listening to radio	Do you listen to the radio at least once a week, less than once a week or not at all?	1 = Not at all 2 = Less than once a week 3 = At least once a week
5.	Age		1 = 15–19 2 = 20–24 3 = 25–29 4 = 30–34 5 = 35–39 6 = 40–44 7 = 45–49
6.	Education	Education level	0 = No formal education 1 = Primary 2 = Secondary 3 = Higher
7.	Religion	Religious affiliation	1 = Christianity 2 = Islam 3 = No religion 4 = Other
8.	Household Wealth Status	Household wealth quintile	0 = Poorest 1 = Poorer 2 = Middle 3 = Richer 4 = Richest
9.	Employment	What is your occupation	1 = Not working 2 = Managerial 3 = Clerical 4 = Sales 5 = Agriculture 6 = Services 7 = Manual
10.	Place of Residence	Type of place of residence	0 = Urban 1 = Rural
11.	Parity	Number of pregnancies reaching viable gestational age	1 = 1 2 = 2 3 = 3 4 = 4 and Above

### Statistical Analysis

The analysis began with computation of proportions of women’s household decision-making capacity among the 30 SSA countries. Secondly, the dataset was appended and this generated a total sample of 47,135. After appending, the overall prevalence and proportions of women’s household decision-making capacity across the socio-demographic characteristics with their significance levels were calculated. Logistic regression analysis was carried out in a hierarchical order. Two hierarchical logistic regression models were built. Model I looked at a bivariate analysis of the three main independent variables, thus, media exposure and the outcome variable. Model II adjusted for media exposure and country by including them in the model together with all the other independent variables. The choice of reference categories for these explanatory variables was similarly informed by propositions of some previous studies (Jensen and Oster, 2009; Ting et al., 2014; Dasgupta, 2019). Logistic regression was employed because our dependent variable (women’s household decision-making capacity) was measured as a dichotomous outcome. Results for the regression analysis were presented as crude odds ratios (COR) and adjusted odds ratios(AOR), with their corresponding 95% confidence intervals (CI) signifying precision and significance of the reported AOR. All frequency distributions were weighted whiles the survey (svy) command in STATA was used to adjust for the complex sampling structure of the data in the regression analyses. All analyses were carried out with STATA version 14.2 for Mac OS.

### Ethical Approval

The DHS surveys obtain ethical clearance from the Ethics Committee of ORC Macro Inc. as well as Ethics Boards of partner organizations of the various countries such as the Ministries of Health. During each of the surveys, either written or verbal consent was provided by the women. Since the data was not collected by the authors of this paper, permission was sought from MEASURE DHS website and access to the data was provided after our intent for the request was assessed and approved on 27th January, 2019.

## Results

### Descriptive Results

#### Proportion of Women With More Capacity to Take Household Decisions in SSA

**FIGURE 1:**
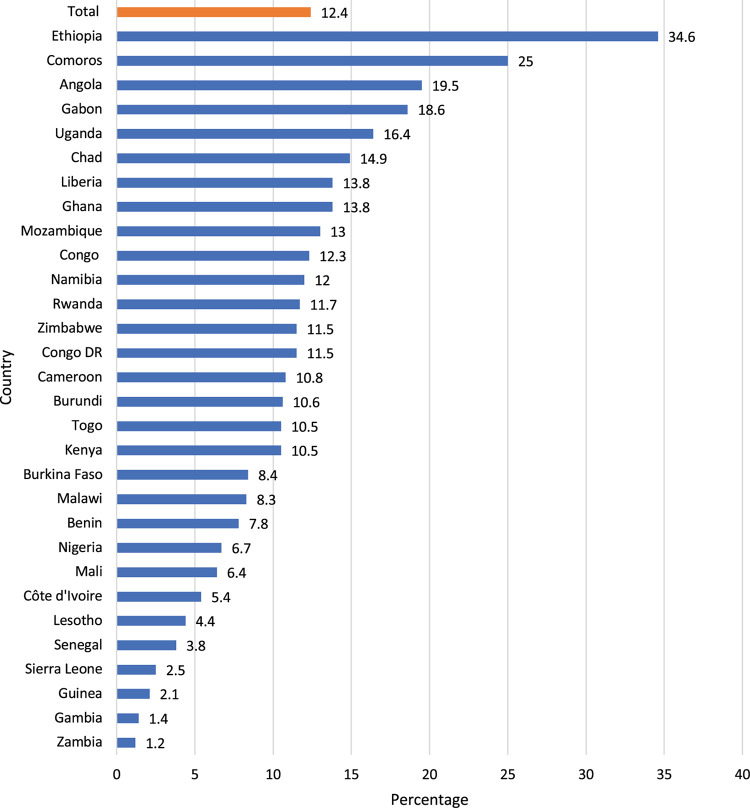
Proportion of women who have the capacity to take household decisions in sub-Saharan Africa (SSA).

Figure 1 presents the distribution of the proportion of women with more capacity to take household decisions in all and each of the 30 SSA countries included in the study. Overall, 12.4% of the women in SSA had more capacity to take household decisions. The proportion of women’s household decision-making capacity ranged from 1.2% in Zambia to 34.6% in Ethiopia.

### Socio-Demographic Characteristics of Respondents

The results on the socio-demographic characteristics showed that 30.7% of the women were aged 20–24 whereas 1.1% were aged 45–49. With educational level, 39.9% had secondary level of education. The majority were Christians (80.8%) while 3.6% had no religious affiliation. Approximately twenty one percent (21.1%) were in the middle and richer wealth quintile while 18.0% were in the poorest wealth quintile. With occupation, 33.4% are not working while 0.7% were in clerical. More than half (53.1%) were in rural areas. Nearly, 36.8% had parity 1 and 13.8% have parity 3 (Table 3).

**TABLE 3:** Socio-demographic characteristics of respondents.

**Variable**	**Weighted N**	**Weighted %**
**Age**		
15–19	6,540	13.9
20–24	14,453	30.7
25–29	11,669	24.8
30–34	7,204	15.3
35–39	4,597	9.8
40–44	2,153	4.6
45–49	520	1.1
**Education level**		
No education	9,125	19.4
Primary	17,582	37.3
Secondary	18,801	39.9
Higher	1,627	3.5
**Religion**		
Christianity	38,063	80.8
Islam	4,808	10.2
No Religion	1,630	3.5
Other	2,633	5.6
**Wealth status**		
Poorest	8,462	18.0
Poorer	9,239	19.6
Middle	9,948	21.1
Richer	9,963	21.1
Richest	9,523	20.2
**Working status**		
Not working	15,732	33.4
Managerial	1,526	3.2
Clerical	338	0.7
Sales	9,467	20.1
Agriculture	13,705	29.1
Services	3,344	7.1
Manual	3,022	6.4
**Residential status**		
Urban	22,117	46.9
Rural	25,018	53.1
**Parity**		
1	17,324	36.8
2	10,084	21.4
3	6,506	13.8
4+	13,221	28.1

Table 4 summarizes the proportions of women who have more capacity to take household decisions across the various mass media channels. Women who read newspaper (14.7%), listened to radio (14.9%) and watched television (16.6%) almost every day had more capacity to take household decisions compared with those who did not read newspaper, listen to radio nor watch television at all, respectively (Table 4).

**TABLE 4:** Mass media exposure and prevalence of women’s household decision-making capacity in sub-Saharan Africa (SSA).

**Variable**	**Sample *N* = 47,135**		**Less Capacity** %	**More Capacity** %	***P*-Value**
	**Weighted N**	**Weighted %**			

**Mass media exposure**					
**Newspaper**					*p* < 0.001
Not at all	35,980	76.3	87.4	12.6	
Less than once a week	5,368	11.4	89.7	10.3	
At least once a week	5,060	10.7	87.2	12.8	
Almost every day	727	1.5	85.3	14.7	
**Radio**					*p* < 0.001
Not at all	18,941	40.2	87.4	12.6	
Less than once a week	8,452	17.9	88.2	11.8	
At least once a week	16,542	35.1	88.0	12.0	
Almost every day	3,199	6.8	85.1	14.9	
**Television**					*p* < 0.001
Not at all	24,637	52.3	88.1	11.9	
Less than once a week	5,291	11.2	88.9	11.1	
At least once a week	10,222	21.7	88.3	11.7	
Almost every day	6,983	14.8	83.4	16.6	

### Multivariable Results

#### Logistic Regression Analysis on the Association Between Mass Media Exposure, Socio-Demographic Characteristics and Women’s Household Decision-Making Capacity in SSA

Women who watched television almost every day [AOR = 1.23; 95%CI = 1.088–1.383] had higher odds of taking household decisions, compared to those who did not watch television at all. On the contrary, women who read newspaper/magazine less than once a week were less likely to take household decisions compared to those who never read newspaper/magazine [AOR = 0.86; CI = 0.774–0.958]. However exposure to radio did not show any statistical significance. In terms of the covariates, age, level of education, wealth index, occupation and parity showed significant associations with women’s household decision-making capacity (Table 5).

**TABLE 5:** Logistic regression analysis on the association between mass media exposure, socio-demographic characteristics and women’s household decision-making capacity in sub-Saharan Africa (SSA).

**Variables**	**Model 1 COR (95%CI)**	**Model 2 AOR (95%CI)**
**Media exposure**					
**Newspaper**		
Not at all	Ref	Ref
Less than once a week	0.788*** [0.715–0.868]	0.861**[0.774–0.958]
At least once a week	0.939[0.851–1.036]	1.009[0.903–1.127]
Almost every day	0.901[0.703–1.154]	0.948[0.732–1.228]
**Radio**		
Not at all	Ref	Ref
Less than once a week	0.982[0.903–1.067]	1.021[0.934–1.115]
At least once a week	0.981[0.916–1.050]	0.956[0.886–1.030]
Almost every day	1.018[0.901–1.149]	0.977[0.860–1.110]
**Television**		
Not at all	Ref	Ref
Less than once a week	0.961[0.871–1.061]	1.089[0.980–1.210]
At least once a week	1.017[0.942–1.098]	1.124*[1.025–1.233]
Almost every day	1.511***[1.386–1.648]	1.227***[1.088–1.383]
**Age**		
15–19		Ref
20–24		1.324***[1.168–1.501]
25–29		1.523***[1.329–1.746]
30–34		1.766***[1.522–2.049]
35–39		2.017***[1.721–2.364]
40–44		2.121***[1.778–2.530]
45–49		2.609***[2.038–3.341]
**Education level**		
No education		Ref
Primary/JHS		1.029[0.946–1.119]
Secondary/SHS		0.956[0.865–1.057]
Higher/tertiary		1.285*[1.040–1.588]
**Religion**		
Christianity		Ref
Islam		0.916[0.807–1.039]
No Religion		1.073[0.926–1.242]
Other		1.068[0.930–1.226]
**Wealth status**		
Poorest		Ref
Poorer		0.886**[0.813–0.965]
Middle		0.904*[0.824–0.992]
Richer		1.749***[1.699–1.868]
Richest		1.771***[1.677–1.877]
**Working status**		
Not working		Ref
Managerial		1.398***[1.185–1.650]
Clerical		1.331[0.930–1.906]
Sales		1.517***[1.395–1.650]
Agriculture		1.153**[1.059–1.256]
Services		1.428***[1.260–1.619]
Manual		1.545***[1.362–1.751]
**Residential status**		
Urban		Ref
Rural		0.780***[0.723–0.841]
**Parity**		
1		Ref
2		1.619***[1.473–1.779]
3		1.832***[1.642–2.045]
4+		2.103***[1.877–2.355]
**Country**		
Angola– 2015–2016		Ref
Burkina Faso– 2010		0.566***[0.409–0.784]
Benin– 2011–2012		0.375***[0.308–0.457]
Burundi– 2015–2016		0.634***[0.534–0.751]
Congo DR– 2013–2014		0.655***[0.565–0.758]
Congo 2011–2012		0.605***[0.530–0.689]
Côte d’Ivoire– 2011–2012		0.277***[0.223–0.344]
Cameroon– 2011		0.646***[0.521–0.802]
Ethiopia– 2016		3.431***[2.346–5.016]
Gabon– 2012		1.006[0.880–1.150]
Ghana– 2014		0.780*[0.641–0.950]
Gambia– 2014		0.111***[0.0352–0.347]
Guinea– 2012		0.180***[0.0791–0.410]
Kenya– 2014		0.734**[0.582–0.927]
Comoros– 2012		2.456[0.923–6.533]
Liberia– 2013		0.846*[0.734–0.976]
Lesotho– 2014		0.278**[0.121–0.637]
Mali– 2012–2013		0.677[0.377–1.218]
Malawi– 2015–2016		0.608***[0.486–0.760]
Mozambique– 2011		1.030[0.863–1.230]
Nigeria– 2013		0.437***[0.336–0.568]
Namibia– 2015		0.736***[0.629–0.861]
Rwanda– 2014–2015		0.758**[0.643–0.895]
Sierra Leone– 2013		0.198***[0.135–0.291]
Senegal– 2010–2011		0.302***[0.167–0.546]
Chad– 2014–2015		0.834[0.575–1.212]
Togo– 2013–2014		0.534***[0.429–0.665]
Uganda– 2016		1.001[0.883–1.134]
Zambia– 2013–2014		0.098***[0.0564–0.170]
Zimbabwe– 2015		0.928[0.688–1.252]
N	47,135	47,135

* p < 0.05, ** p < 0.01, *** p < 0.001, Ref, reference; COR, crude odds ratio; AOR, adjusted odds Ratio.

## Discussion

Women’s decision-making capacity is crucial, not only for human right purposes but other wellbeing consequences such as reproductive health and holistic wellbeing (Sell and Minot, 2018). Similarly, the importance of mass media in behavioral change agenda such as empowering women is of no doubt (Ali et al., 2011; Letamo and Navaneetham, 2015). Consequently, we investigated the influence of mass media (newspaper, television and radio) exposure on women’s household decision-making capacity in 30 countries in SSA. Such an enquiry is of much essence in enhancing the livelihood of women in the wake of synergistic efforts at the global level toward ensuring active participation of women in social, political and economic spheres of life (World Bank Group, 2019).

Results showed that women who watched television almost every day had more capacity to take household decisions compared to women who were not watching television at all. This is essential because the observation made in our study possibly implies that audio-visual media platforms (i.e., television) works better for a greater segment of women in SSA. Television, for instance, has been reported to be effective and more receptive in communicating health information to women than other media options (Lyu et al., 2014). Visuals on the television are attractive and very impactful to the masses (Botchway, 2019). In contrast to our findings on the association between exposure to television and women empowerment, we observed that women who read newspaper less than once a week were less likely to be empowered compared to those who never read newspaper. We also found no statistically significant association between the frequency of listening to radio and women empowerment. Although previous studies have identified both radio and newspaper to be playing roles in women empowerment (Narayana and Ahamad, 2016; Dasgupta, 2019) there are several pathways we can explain these contrary findings. For instance, in relation to newspaper, findings of previous studies, have indicated that in SSA the impact of newspaper on women empowerment depends on the level of literacy and analytical skills which have been to be low within the sub-region (Bhattacharya, 2016). Again, women empowerment campaigns have been regarded as dependent on exposure to television compared to newspaper because newspapers are often self-selective in coverage in favor of literates and people who can afford (Bala, 2017). The insignificant association between exposure to radio and women empowerment is a finding that needs further research. Notwithstanding, the possible reasons for this insignificant finding could be related to the characteristics of the study population in terms of those who are exposed and not exposed to radio.

Relative to Angola, women from Ethiopia and Zambia had the highest and lowest odds of taking household decisions, respectively. Decision-making prospects of women cannot be untangled from the social structures, policy frameworks and political commitment at the local, national or regional level (Thandar et al., 2019). This variation observed across countries may be rooted in some of the aforementioned factors, indicating that every society or nation seeking to enhance women’s empowerment status can influence the available structures and frameworks in a direction that can guarantee the desired results. Probing and finding answers to what is happening differently in Ethiopia from Zambia or Angola can be a great start for pinpointing the requisite and feasible interventions to put in place. For instance, one pivotal strategy for empowering women in Ethiopia since 2012 has focused on enhancing women’s financial and entrepreneurial capacity (World Bank Group, 2019). Due to the war in 2002, Angola is an emerging country as far as women empowerment policies and strategies are concerned and the initiatives in Angola may need some considerable time to manifest. Some of the recent initiatives include the National Development Plan for 2013–2017, the Family Code and Power Africa (White, 2017).

Women aged 45–49 years had the highest odds of taking household decisions compared to those aged 15–19. The former age brackets constitute women at the end of their reproductive life and may have finished giving birth. All things being equal, women in 45–49 year category are likely to have more maternal experience. This assumption has been identified as an enabler to decision-making prospects (Yuill et al., 2020). However, a United States based study was inconclusive about how age relates with decision making (Bruine de Bruin et al., 2012). Yount et al. (2018) espoused that empowerment condition for women worsens if they marry at a tender age. A number of studies including Head et al. (2015) and Akram (2018) came to a similar conclusion that older women tend to be more empowered when it comes to decision-making compared with young women. Considering this age results vis-a-vis earlier reports, it is reasonable to infer that age provides some leverage for women thereby enhancing their decision-making capacity within households and communities in which they live. The current result is likely because women who are advanced in age may command some level of respect/recognition from socio-cultural perspective. Azra Batool et al. (2018) also espoused that advanced age benefits the entire family in addition to affecting women’s lives positively. For instance, children of empowered women tend to receive better education.

Women with higher education had the highest odds of taking household decisions. This result strengthens the consistent evidence in literature about the importance of education in enhancing women’s decision-making capacity within the household, society and national levels (Krause et al., 2016; Narayana and Ahamad, 2016; Sell and Minot, 2018). The same outcome has been reported from Sri Lanka, Nepal, and Bangladesh (Senarath and Gunawardena, 2009). Education prepares women to be confident in decision making and also equips them to participate effectively in political and developmental agenda (Saravanakumar and Varakumari, 2019). If a woman can take up political roles and influence developmental agenda, ability to decide will not be a challenge. At least, ensuring that every female attains secondary level of education can go a long way to advance women empowerment in all facets of their lives (Sell and Minot, 2018).

Other results revealed that richest women were more likely to take household decisions unlike the poorest. Being rich exerts some level of power in influencing choices and decisions (Shahbaz et al., 2017). Further, richest women are likely to have higher education relative to the poor due to cost associated with higher education (Whistle, 2019). Akram (2018) suggested that persons from poor households usually think negatively about women whilst the women from same wealth quintile usually experience domestic violence and are often not consulted on matters affecting families and/or households compared to their counterparts from rich families. Women who were working were more likely to take household decisions irrespective of the type of occupation they were engaged in. The positive role of employment in ensuring women’s empowerment, including their household decision-making capacity has been noted in literature (Head et al., 2015; Salem et al., 2017). Similar to a study in three Asian countries (i.e., Nepal, Bangladesh, and Sri Lanka), women who were employed and earned income had increased decision making power (Senarath and Gunawardena, 2009). One pathway by which employment enhances household decision-making capacity is the likelihood to reduce the economic dependency of women on their partners. Employment generates personal income which enhances self-confidence and esteem (Al-Amin and Chowdhury, 2018). Therefore, increasing employability skills and providing job avenues for women can augment women empowerment efforts as maintained by some scholars that women who engage in paid jobs have a ‘voice’ in decision making (Letamo and Navaneetham, 2015; World Bank Group, 2019). This assertion suggests a close relation between economic position of women and their empowerment prospects (Shettar and Rajeshwari, 2015). Therefore, creating employment opportunities for women is a promising strategy for enhancing their household decision-making capacity. Compared to urban women, those in rural settings had lower odds of taking household decisions. Sell and Minot (2018) summarized that women in rural settings are usually less empowered due to limited engagement in value added economic activities. Similar report has emerged from South Asia (Sharma and Shekhar, 2015), suggesting that this finding is not only peculiar to SSA. The consistency in evidence possibly indicates that some opportunities exist in urban areas that augment women’s household decision-making capacity prospects compared to rural locations.

### Strength and Limitations

Using same indicators for measuring women’s household decision-making capacity across 30 countries in SSA, the study has offered insights on the association between mass media exposure and household decision capacity. The wide coverage and rigor of the analytical procedure have enhanced the prospects of generalizing the findings to other contexts where empowerment status of women are to be improved. However, due to the cross-sectional nature of the study design, causal inference cannot be drawn from current outcomes. Also, we were unable to control for the effects of the kind or nature of media contents women were exposed to due to the secondary data used.

## Conclusion

The study has demonstrated that exposure to television plays a significant role in women’s decision making capacity within households. A number of policies and guidelines exist; however, much concerted efforts and collaborations are required of governments, human right organizations, and civil societies to ensure that the policies are well implemented. Mass media (television) could be utilized in a very affective and persuasive manner in enhancing the household decision-making capacity of women. Television, can therefore be used to communicate to women and the general populace on the relevance and ways of achieving women’s household decision-making capacity in SSA and other low and middle-income countries. Interest groups that require greater attention are women with less exposure to television as well as women in their early reproductive age, the poor, women who are not working and rural residents. Future research could explore if mass media exposure can enhance men’s support for women’s household decision-making. Also, similar studies can investigate the kinds of television programs that are more able to improve women’s household decision-making capacity in SSA.

## Data Availability Statement

Publicly available datasets were analyzed in this study. This data can be found here: https://dhsprogram.com/data/available-datasets.cfm.

## Ethics Statement

The studies involving human participants were reviewed and approved by Ethics Committee of ORC Macro, Inc., as well as Ethics Boards of partner organizations. The patients/participants provided their written informed consent to participate in this study.

## Author Contributions

A-AS conceived and designed the study and analyzed and/or interpreted the data. A-AS, EKA, EbA, BA, AO, JH, ErA, and TS drafted the manuscript. A-AS, EKA, JH, EbA, BA, AO, ErA, FS, VT, and TS revised the manuscript critically for important intellectual content. EbA and ErA proofread the manuscript. All authors have read and approved the final manuscript.

## Conflict of Interest

The authors declare that the research was conducted in the absence of any commercial or financial relationships that could be construed as a potential conflict of interest.

## Abbreviations

SSA: sub-Saharan Africa
COR: crude odds ratios
AOR: adjusted odds ratios
PSU: primary sampling units
EA: enumeration areas
DHS: Demographic and Health Survey
CI: confidence interval.
